# A bivariate genome-wide association study identifies ADAM12 as a novel susceptibility gene for Kashin-Beck disease

**DOI:** 10.1038/srep31792

**Published:** 2016-08-22

**Authors:** Jingcan Hao, Wenyu Wang, Yan Wen, Xiao Xiao, Awen He, Xiong Guo, Tielin Yang, Xiaogang Liu, Hui Shen, Xiangding Chen, Qing Tian, Hong-Wen Deng, Feng Zhang

**Affiliations:** 1 Key Laboratory of Trace Elements and Endemic Diseases of National Health and Family Planning Commission, School of Public Health, Health Science center, Xi’an Jiaotong University, Xi’an, P. R. China; 2 Key Laboratory of Biomedical Information Engineering of Ministry of Education, and Institute of Molecular Genetics, School of Life Science and Technology, Xi’an Jiaotong University, Xi’an, P.R. China; 3 Department of Biostatistics and Bioinformatics, Tulane University School of Public Health and Tropical Medicine, New Orleans, Louisiana, USA; 4 Center for Bioinformatics and Genomics, Tulane University, New Orleans, Louisiana, USA; 5 Laboratory of Molecular and Statistical Genetics, College of Life Sciences, Hunan Normal University, Changsha, P. R. China

## Abstract

Kashin-Beck disease (KBD) is a chronic osteoarthropathy, which manifests as joint deformities and growth retardation. Only a few genetic studies of growth retardation associated with the KBD have been carried out by now. In this study, we conducted a two-stage bivariate genome-wide association study (BGWAS) of the KBD using joint deformities and body height as study phenotypes, totally involving 2,417 study subjects. Articular cartilage specimens from 8 subjects were collected for immunohistochemistry. In the BGWAS, *ADAM12* gene achieved the most significant association (rs1278300 p-value = 9.25 × 10^−9^) with the KBD. Replication study observed significant association signal at rs1278300 (p-value = 0.007) and rs1710287 (p-value = 0.002) of A*DAM12* after Bonferroni correction. Immunohistochemistry revealed significantly decreased expression level of ADAM12 protein in the KBD articular cartilage (average positive chondrocyte rate = 47.59 ± 7.79%) compared to healthy articular cartilage (average positive chondrocyte rate = 64.73 ± 5.05%). Our results suggest that *ADAM12* gene is a novel susceptibility gene underlying both joint destruction and growth retardation of the KBD.

Kashin-Beck disease (KBD) is a serious osteoarthropathia^1^,^2^. The KBD mainly manifests as joint pain, joint destruction and growth retardation. It is mainly prevalent at defined areas of China, Russia and North Korea. In China, there are more than 2.5 million KBD patients, and about 30 million people living in the KBD prevalent areas are at the risk of acquiring the diseases^3^. According to disease severity, the KBD is clinically classified into three grades, grades I, II and III. Comparing with the grade I KBD patients, the grade II and III KBD patients have serious multi-joint deformities and distinctive growth retardation, for instance, a short stature.

Extensive pathogenetic studies of the KBD have been conducted. However, the molecular mechanism of the KBD remains unclear, which results in the lack of effective treatments for the KBD today. Recently, a large genetic study invoking 10,823 subjects from 1,361 Han Chinese families observed a significant familial clustering of the KBD patients^4^, indicating a strong genetic determinant of the KBD. Several susceptibility genes have been identified for the KBD^5^,^6^. However, the reported susceptibility genes provide only a limited explanation for the KBD risks, suggesting the existence of unknown susceptibility genes. The genetic basis of the KBD urgently needs further studies.

In all previous association studies, the KBD was treated as a qualitative trait by using joint destruction as a study phenotype. However, the growth retardation is another important outcome of the disease. To the best of our knowledge, no genetic study including the growth retardation in the analysis of the KBD samples has been carried out by now. This may have led to a neglection of the susceptibility genes involved in the growth retardation of the KBD. Bivariate association studies, which simultaneously consider two disease phenotypes can help to address this issue. They are capable to identify pleiotropic genes, and to alleviate multiple testing problems caused by analyzing different disease phenotypes separately. Bivariate association studies have been successfully used in the susceptibility mapping of complex diseases^7^,^8^. Furthermore, Saint-Pierre *et al*. suggest that bivariate analysis performed well in the GWAS of correlated disease phenotypes^9^.

Motivated by the lapses in the current susceptibility gene studies of the KBD, we performed a two-stage bivariate genome-wide association study (BGWAS) of the KBD for the data, which totally involved 2,417 study subjects. Immunohistochemistry of the KBD articular cartilage and the healthy articular cartilage was also performed to evaluate the functional relevance of identified genes with the KBD.

## Results

### Bivariate association analysis

A structure analysis found that all the study subjects were clustered together as one homogeneous population. The genomic control inflation factor λ, calculated by EIGENSTRAT software was 1.03, indicated a negligible impact of population stratification on our study results.

**Figure 1:**
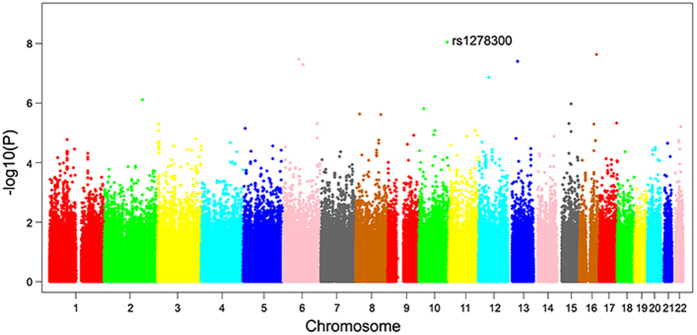
Manhattan plot of the genome-wide association study results. X-axis shows chromosomal positions. Y-axis shows –log10 (p-value).

A total of 532,894 single-nucleotide polymorphisms (SNPs) passed a quality control and were used for the BGWAS. It presents a good match between the distribution of observed p-values and those expected by chance. Manhattan plot of the BGWAS results across the genome is presented by Fig. 1. Across the whole genome, only ADAM12 (disintegrin and metalloproteinase domain-containing protein 12) gene achieved significant association (rs1278300 p-value = 9.25 × 10^−9^) with KBD.

### Replication study

The significant SNP rs1278300 of the ADAM12 in the BGWAS was selected for replication study. To avoid missing association signals, six additional SNPs within the ADAM12 were also selected for replication study. Table 1 summarizes the replication association analysis results for the ADAM12. We observed that rs1278300 (p-value = 0.007) and rs1710287 (p-value = 0.002) were significantly associated with the KBD after Bonferroni correction. rs11244772 also showed a suggestive association signal (p-value = 0.028) with the KBD which was however not significant after Bonferroni correction (Table 1). ADAM12 encodes disintegrin and metalloproteinase domain-containing protein 12, which is implicated in a variety of biological processes involving cell-cell and cell-matrix interactions. Both the BGWAS and the replication study observed significant association between the KBD and the ADAM12, which supported the functional relevance of the ADAM12 with the KBD.

### Immunohistochemistry

**Figure 2:**
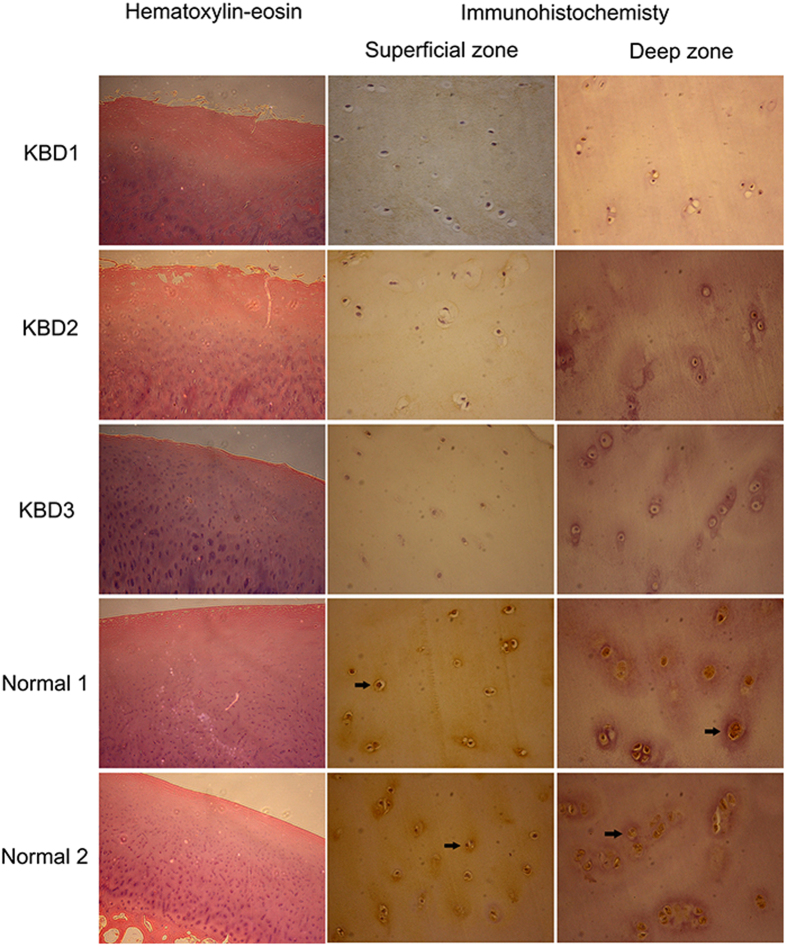
The images of immunohistochemistry of the KBD articular cartilage and the healthy one.

To assess the functional relevance of the ADAM12 with the KBD, we further compared the protein levels of the ADAM12 in the articular cartilages of five KBD patients and three healthy subjects using immunohistochemistry. We observed that the expression level of the ADAM12 protein in the KBD articular cartilage was significantly lower than that in the healthy one (Fig. 2). The average percentage of the ADAM12 positive chondrocytes of five KBD patients was 47.6 ± 7.8% (mean ± SD), while in the samples of three healthy subjects it was 64.7 ± 5.0% (mean ± SD).

## Discussion

Previous susceptibility gene studies of the KBD have focused on joint destruction, which may have cause the omission of the causal genes underlying behind the growth retardation of the KBD. In this study, we performed a two-stage genome-wide bivariate association study of the KBD. To the best of our knowledge, this is the first susceptibility gene study of the KBD, which simultaneously takes into account the joint destruction and the growth retardation of the KBD. The BGWAS observed that the ADAM12 gene achieved the most significant association signal for the KBD. The replication association study further confirmed the association between the ADAM12 and the KBD in the independent validation sample. Immunohistochemistry showed that the expression level of the ADAM12 protein in the KBD articular cartilage was lower than that in the healthy one, supporting the functional relevance of the ADAM12 with the KBD. Our study results suggest that the ADAM12 is a novel susceptibility gene with pleiotropic effects on the joint destruction and the growth retardation of the KBD.

The ADAM12 gene encodes a member of the matrix metalloproteinases. *In vivo*, the ADAM12 binds insulin growth factor binding protein-3 (IGFBP-3)^10^. The ADAM12 is implicated in a variety of biological processes, such as muscle development and neurogenesis^11^. The expression of the ADAM12 can be used as a maternal serum marker for pre-natal development^12^. No implication of the ADAM12 in the skeletal growth retardation of the KBD has been reported so far. However, previous studies demonstrated that the ADAM12 involved in the skeletal growth and development^12^. The ADAM12 has a regulatory role in both osteoblasts and osteoclasts^13^,^14^. For instance, it involves in human bone formation and osteoclast differentiation^15^, and it is also capable of stimulating longitudinal bone growth in the ADAM12 transgenic mice by modulating chondrocyte proliferation and maturation^15^. The ADAM12 transgenic mice with a high expression level of ADAM12 protein exhibited increased longitudinal bone growth^15^, which is consistent with our immunohistochemistry results. In this study, we observed a decreased expression of the ADAM12 protein in the articular cartilage of the KBD patients exhibiting short stature. It is reasonable to infer that dysfunction of the ADAM12 may be involved in the skeletal growth retardation of the KBD.

The ADAM12 is also implicated in the pathogenesis of osteoarthritis. Multiple genetic association studies have observed a significant association between the ADAM12 and the osteoarthritis^16^,^17^,^18^. A significant correlation was also found between serum ADAM12 protein level and radiographic grades of knee osteoarthritis^19^. Additionally, the ADAM12 was reported to involve in synovial inflammation^20^, which plays an important role in the progression of osteoarthritic joint degradation. Based on the study results of previous and our studies, we suggest that the ADAM12 is a susceptibility gene of the KBD. Further studies are needed to confirm our finding and reveal the potential molecular mechanism of the ADAM12 involve in the development of the KBD.

There are some limitations in this study. First, we recruited 90 KBD patients and 1,627 healthy controls as the study samples of the BGWAS. To ensure the statistical power of the BGWAS, we used extreme phenotype sampling. It has been demonstrated that extreme phenotype sampling is capable of largely reducing sample sizes needed for the analyses without a significant loss of statistical power^21^. All of the 90 KBD patients exhibited representative KBD phenotypes including serious joint pain, shortened phalanges, shortened humeri, serious multi-joint deformities and short stature. Using the KBD patients with similar extreme phenotypes should enrich causal genetic variants and, therefore, increase the statistical power of the BGWAS. Additionally, we applied bivariate analysis, which should be more powerful than univariate analysis^22^,^23^. For complex diseases, the bivariate association analysis is capable of incorporating the pathogenetic information of multiple disease-related traits, and alleviating the issue of multiple testing problems. It can help to improve the power and precision to locate and evaluate the genetic effects of the causal genes^22^,^23^. The results of the replication study using an independent validation sample and immunohistochemistry are consistent with the results of the BGWAS, which supported the accuracy of the BGWAS. Second, it should also be noted that there was a significant age difference between the KBD and the control samples in the BGWAS. To eliminate the potential impact of the age on the BGWAS results, raw phenotypes were adjusted for the age as a covariate before the association analysis.

In summary, we conducted the first BGWAS study of KBD. Our study results suggest that the ADAM12 gene is a novel susceptibility gene underlying both the joint destruction and the growth retardation of the KBD. This study provides a new insight into the pathogenesis of the KBD.

## Materials and Methods

### Ethics Statement

This study was approved by the Institutional Review Board of Xi’an Jiaotong University (Project Number: 2014008). The study was conducted in accordance with the principles of Helsinki Declaration. A written informed consent was obtained from all the subjects.

### Human subjects

To ensure the statistical power of bivariate association study, extreme phenotype sampling was applied. Clinical data of each participant was recorded by nurse-administered questionnaire, including self-reported ethnicity, lifestyle characteristics, health status, family and medical histories. All the study subjects underwent careful clinical examination and radiography of the skeletal system. According to the KBD clinical diagnosis criteria of China (WS/T207-2010), the joint destruction of each KBD patient was scored according to the extents of joint pain, restricted movement of elbow and knee joints, enlarged finger joints, shortened phalanges and humeri. The growth retardation of the KBD was measured by body heights in this study. Fifty nine grade II and 31 grade III genetically unrelated KBD patients (50 males and 40 females) with a serious multiple-joint destruction and the growth retardation were previously recruited for the genetic studies of the KBD from Linyou county of Xi’an city of Shaanxi Province in China^24^. Their average height and age were 148.8 ± 15.7 cms and 59.1 ± 8.4 years, respectively. As controls, 1,627 genetically unrelated healthy subjects (802 males and 825 females), originally recruited for osteoporosis studies^25^, were used. Their average height and age were 164.2 ± 8.2 cms and 34.5 ± 13.2 years, respectively. For the replication study, another independent sample containing 700 subjects was recruited from Yongshou county and Bin county of Xi’an city of Shaanxi Province in China. Of them, 403 subjects were diagnosed as having the KBD, including 255 grade I, 129 grade II and 19 grade III KBD patients (Table 2). We excluded the subjects with genetic bone and cartilage diseases, primary osteoarthritis, rheumatoid arthritis, as well as family history of articular disorders (including first and second degrees relatives). Five ml of peripheral blood was drawn from each participant.

### Genotyping and quality control

DNA specimens were extracted from the peripheral blood leukocytes using the E.Z.N.A. Blood DNA Midi Kit (Omega Bio-tek, Norcross, USA) or Puregene DNA isolation kit (Gentra Systems, Minneapolis, USA) following the manufacturers’ protocol. Affymetrix Genome Wide Human SNP Array 6.0 (Affymetrix, Santa Clara, CA, USA) was applied for genotyping. Fluorescence intensities were quantified by Affymetrix 30007G scanner (Affymetrix). Data management and quality control were performed by AffymetrixH GeneChipH Command ConsoleH software. All the GWAS samples passed a genetic relatedness check based on pairwise identity by state testing (implemented by PLINK)^26^. No duplicated and genetically related samples were detected. The average call rate of the BGWAS samples was 98.92%. For quality control, the SNPs with Hardy-Weinberg Equilibrium testing p*-*value < 0.001, call rates <98.00% and minor allele frequencies <0.01 were excluded.

### Bivariate association analysis

Structure analysis (http://pritchardlab.stanford.edu/structure.html) was used to evaluate the genetic background of the BGWAS samples^27^. EIGENSTRAT software (http://genetics.med.harvard.edu/reich/Reich_Lab/Software.html) was used to calculate the genomic control inflation factor λ^28^. To eliminate the potential impacts of the age and the sex on the BGWAS results, the raw phenotypic values were adjusted for the age and the sex as covariates before the bivariate association analysis. Using additive genetic model, a bivariate liner regression model implemented by R package (http://www.r-project.org) was applied for the BGWAS of the KBD^29^. The analysis procedure of the BGWAS has been detailed in the previous study^29^. Briefly, the bivariate association analysis was conducted using the bivariate liner regression model, defined as *Y*_*i*_ = *μ* + *βX*_*i*_ + *ε*_*i*_. *Y*_*i*_denotes the vector of two phenotypes for an individual *i; μ* denotes the ground mean vector; *β* denotes the regression coefficient vector of the target SNP; *X*_*i*_denote the genotype score of the target SNP for the individual *i; ε*_*i*_ denotes the residual random effect vector following a multivariate normal distribution with mean zero. Likelihood ratio tests were conducted to evaluate the significance of *β* using the “lm” command of R (https://www.r-project.org/). The raw p-value of each SNP was reported here.

### Replication study

The significant SNP rs1278300 of the ADAM12 in the BGWAS was selected for replication study in the independent validation sample of 700 study subjects. To avoid missing association signals, six additional SNPs within the ADAM12, were also selected for replication study. Sequenom MassARRAY platform (Sequenom, San Diego, CA, USA) was applied for the genotyping according to the manufacturer’s protocol. The MassARRAY Assay Design 3.1 (Sequenom, San Diego, CA, USA) was used to design the primers of polymerase chain reaction experiment. Sequenom MassArray TYPER 4.0 (Sequenom, San Diego, CA, USA) was used for SNP calling. The quality control and the association analysis were conducted using the same approaches as used in the BGWAS.

### Immunohistochemistry

The articular cartilage specimens were collected from five KBD patients undergoing total knee replacement surgery, and three healthy control subjects undergoing knee amputation. The paraformaldehyde-fixed articular cartilage tissues of the KBD and the healthy subjects were rinsed with phosphate buffered saline (PBS), decalcified and embedded in paraffin. The paraffin-embedded cartilage tissues were sectioned (5~8 μm thick), and placed on glass slides. For histochemistry, the cartilage tissue slides were dewaxed in xylene, hydrated with graded ethanol, and stained respectively by hematoxylin-eosin and Safranin O. For immunohistochemistry, the dewaxed and hydrated cartilage sections were treated with 3% hydrogen peroxide solution for 10 mins, rinsed with PBS, and incubated with rabbit polyclonal anti-ADAM12 antibody (1:50 working dilution, Abcam plc, MA, UK) at 4 °C overnight. The cartilage sections were then incubated with secondary antibody (Zhongshan Golden Bridge Biotechnology, China) at 37 °C for 15 mins, exposed to Streptavidin-Horseradish Peroxidase at 37 °C for 15 mins, and stained with diaminobenzidine. The percentage of the ADAM12 positive chondrocytes in 1,000 chondrocytes was calculated for each cartilage specimen.

## Additional Information

**How to cite this article**: Hao, J. *et al*. A bivariate genome-wide association study identifies ADAM12 as a novel susceptibility gene for Kashin-Beck disease. *Sci. Rep.* **6**, 31792; doi: 10.1038/srep31792 (2016).

## Figures and Tables

**Table 1:** Association analysis results of the ADAM12 in the BGWAS and the replication study.

SNP	Location	Allele	BGWAS p-value			Replication p-value		
			JD	GR	Bivariate	JD	GR	Bivariate
rs1278300	126102615	C/T	1.99 × 10^−6^	0.014	9.25 × 10^−9^	0.022	0.016	0.007
rs11244772	126022727	A/G	0.047	0.033	0.025	0.042	0.032	0.028
rs1710287	126052621	A/G	0.030	0.068	0.041	0.019	0.013	0.002
rs12415903	126074317	C/T	0.012	0.066	0.036	0.299	0.389	0.219
rs1380439	126079541	C/T	0.013	0.073	0.043	0.479	0.068	0.189
rs12217845	126250136	C/T	0.087	0.018	0.030	0.823	0.568	0.680
rs7902734	126264332	A/G	0.093	0.012	0.031	0.801	0.477	0.614

Note: BGWAS denotes the bivariate genome-wide association study; JD denotes the joint destruction; GR denotes the growth retardation.

**Table 2:** Basic characteristics of the study subjects.

	N	Age^a^ (years)	Sex (male/female)	Height^a^ (cm)	Weight^a^ (Kg)
BGWAS					
KBD	90	59.14 ± 8.40	50/40	148.82 ± 15.70	47.01 ± 8.99
Health subjects	1627	34.49 ± 13.24	802/825	164.25 ± 8.16	60.12 ± 10.48
Replication					
KBD	403	59.25 ± 8.82	169/234	154.26 ± 10.44	52.11 ± 10.40
Health subjects	297	56.30 ± 10.58	112/185	159.78 ± 8.19	57.60 ± 10.83

^a^Denotes mean ± SD
